# Melatonin and Cancer Hallmarks

**DOI:** 10.3390/molecules23030518

**Published:** 2018-02-26

**Authors:** Wamidh H. Talib

**Affiliations:** Department of Clinical Pharmacy and Therapeutics, Applied Science Private University, Amman 11931-166, Jordan; w_talib@asu.edu.jo; Tel.: +9-626-560-999 (ext. 1141)

**Keywords:** melatonin, cancer hallmarks, anticancer, angiogenesis, metastasis, immune evasion

## Abstract

Melatonin is a natural indoleamine produced by the pineal gland that has many functions, including regulation of the circadian rhythm. Many studies have reported the anticancer effect of melatonin against a myriad of cancer types. Cancer hallmarks include sustained proliferation, evading growth suppressors, metastasis, replicative immortality, angiogenesis, resisting cell death, altered cellular energetics, and immune evasion. Melatonin anticancer activity is mediated by interfering with various cancer hallmarks. This review summarizes the anticancer role of melatonin in each cancer hallmark. The studies discussed in this review should serve as a solid foundation for researchers and physicians to support basic and clinical studies on melatonin as a promising anticancer agent.

## 1. Introduction

**Figure 1:**
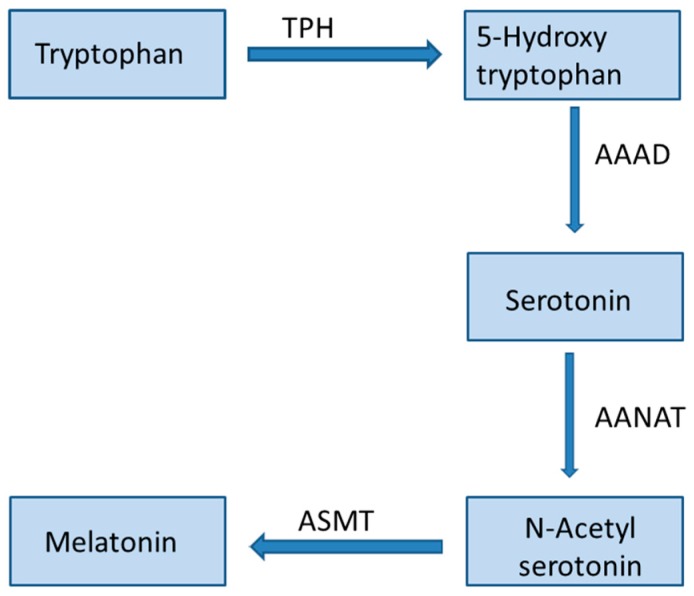
Melatonin biosynthetic pathway. TPH: tryptophan hydroxylase; AAAD: aromatic l-amino acid decarboxylase; AANAT: arylalkylamine *N*-acetyltransferase; ASMT: acetylserotonin *O*-methyltransferase.

Melatonin (*N*-acetyl-5-methoxytryptamine) is an indoleamine produced by humans and other animals in response to darkness. The main producer of this hormone is the pineal gland and it is also produced in several organs like the gastrointestinal tract, skin, retina, and bone marrow [1,2]. Chemically, melatonin is an indolic compound derived from the amino acid tryptophan and it has lipophilic properties. The biosynthetic pathway of melatonin is summarized in Figure 1.

In addition to the dark-light cycle, the levels of this hormone are controlled by seasons, gender, age, and physiological conditions [3]. Circadian rhythm monitoring is only one of the many functions of melatonin, which also has immunomodulatory, anti-inflammatory, antioxidant, vasoregulation, and oncostatic activities [4,5,6,7,8].

The relationship between melatonin and cancer has been investigated during the last 80 years and several epidemiological studies support the anticancer activities of this hormone [9,10]. Experimental studies showed that the normally elevated levels of melatonin at night help in the organization of homeostatic metabolic rhythms of targeted organs and systems [11]. Accordingly, circadian rhythm disruption could be one of the contributing factors in cancer development and progression [12].

Cancer is the second cause of death globally and recent reports have revealed that by 2025, an increase of 19.3 million new cases per year is expected [13]. A study in USA estimated 1,685,210 new cancer cases and 595,690 cancer deaths in 2016 [14].

Surgery, radiotherapy and chemotherapy are the main conventional anticancer therapies. However, the limited efficacy of these therapies and their serious side effects have encouraged the search for new anticancer agents based on natural products and herbal extracts as single therapies or in combination with other agents [15,16,17,18,19].

The concept of cancer hallmarks was first proposed in 2000 by Hanahan and Weinberg. The hallmarks summarized the biological capabilities required by cells to start the conversion process from normal cells to cancer cells [20]. In 2000, Hanahan and Weinberg proposed six hallmarks. These hallmarks were expanded to eight in 2011 by the same scientists. The eight hallmarks are: sustained proliferation, growth suppression evasion, tissue invasion and metastasis, replicative immortality, angiogenesis induction, cell death resistance, altered cellular energetics, and immune evasion [21].

During the last decades, many studies have explained the anticancer activities of melatonin. This hormone can exert its anticancer effect through receptor-dependent and receptor-independent mechanisms [10]. Melatonin has two types of receptors that belong to the G-protein superfamily and are involved in mediating the anticancer effect by inhibiting linoleic acid uptake [22]. The receptor-independent anticancer effects of melatonin involve diverse mechanisms including apoptosis induction, angiogenesis inhibition, immune evasion, and altered cancer metabolism [23,24]. Additionally, melatonin was used as adjuvant therapy to augment the anticancer effects of different agents [25,26,27].

### 1.1. Melatonin Metabolism

The liver is the main site for circulating melatonin metabolism. The first step is mediated by cytochrome P_450_ mono-oxygenases and involves hydroxylation of melatonin in the C6 position. This step is followed by conjugation of the product with sulfate to produce 6-sulfatoxymelatonin [28]. Melatonin metabolism also involves non-enzymatic reactions taking place in all cells and extra-cellularly. In these reactions, melatonin is converted to cyclic-3-hydroxymelatonin after scavenging 2 hydroxyl radicals. In non-hepatic tissue, *N*^1^-acetyl-*N^2^*-formyl-5-methoxykynuramine (AFMK) is the central metabolite of melatonin oxidation. The next step in this pathway (kynuric pathway) is the conversion of AFMK to AMK (*N*^1^-acetyl-5-methoxykynuramine) [22].

### 1.2. The Light/Dark Cycle and Regulation of Melatonin Release

Pineal melatonin production is regulated by the daily light/dark cycle. High concentrations of melatonin are produced at night and levels decrease during the day. The link between the pineal gland and light starts in the retina which receives light and send signals through the retinohypothalamic tract to the suprachiasmatic nucleus which is a circadian oscillator in the brain [29]. From the suprachiasmatic nucleus, fibers pass through the paraventricular nucleus, medial forebrain bundle, reticular formation and finally terminate in the spinal cord. From the spinal cord, fibers project to the superior cervical ganglion which sends postganglionic neurons to fibers innervating the pineal gland and regulating the process of melatonin production [30]. During the light hours of the day, neurons in the suprachiasmatic nucleus are highly activated and send inhibitory signals to the pineal gland. At night, the suprachiasmatic nucleus is inhibited and no inhibitory signals are sent to the pineal gland which in turn starts melatonin production [31].

The blood levels of melatonin are controlled by the rate of synthesis and the rate of peripheral degradation (mainly in the liver) [32]. Exposure to light and disruption of the blood melatonin levels is associated with many diseases, including cancer [33]. Serum melatonin levels ≤39.5 pg/mL causes a 15-fold increase in breast cancer risk compared with subjects with higher serum levels of melatonin [34]. In another study, high levels of melatonin’s primary metabolite 6-sulphatoxymelatonin (aMT6s) in urine is associated with a lower risk of breast cancer [35]. In men, levels of urinary aMT6s below the median increase the risk of prostate cancer 4-fold compared with subjects having normal levels [36]. Additionally, high serum melatonin levels are associated with low prostate cancer incidence [37]. A similar association for ovarian cancer was also observed in women [38].

The objective of this review is to summarize the research on the anticancer effects of melatonin and to discuss the mechanisms activated by this hormone to inhibit each cancer hallmark.

## 2. Melatonin and Cancer Hallmarks

### 2.1. Role of Melatonin in Genomic Instability

Genomic instability is one of the critical steps in cancer initiation and progression. It provides a selective growth advantage for cancer cells over neighboring cells [13]. Throughout the cell cycle of normal cells, the integrity of the genome is protected by checkpoints. During cancer development, the presence of aneuploidy nuclei (having an abnormal number of chromosomes) indicates the failure of one or more of these cell cycle check points [39]. Cell cycle check points are regulated by proteins that either encourage (oncogene products) or inhibit cell division (tumor suppressor gene products). The activity of these proteins is normally altered in cancer cells to encourage uncontrolled cell growth [15]. Five targets were suggested to alter genomic instability. These targets are: DNA damage prevention, stimulation of DNA repair system, deficient DNA repair targeting, targeting impaired clustering of centrosome, and telomerase activity inhibition [13].

The antioxidant activity of melatonin can protect against DNA damage either by scavenging reactive oxygen species or by stimulating the DNA repair system. In one study, melatonin (50 µM) and its direct metabolite *N*^1^-acetyl-*N*^2^-formyl-5-methoxykynuramine caused a reduction in DNA damage induced by exposure to hydrogen peroxide. Additionally, this treatment caused chemical inactivation of hydrogen peroxide preventing its damaging effects [40]. In another study, melatonin protect against UV radiation damage by enhancing gene expression of antioxidative enzymes and preventing the formation of 8-hydroxy-2′-deoxyguanosine (DNA-base-oxidized intermediate) [41]. Radiation-induced oral mucositis was inhibited in rats treated with melatonin (45 mg/day) for 21 days [42]. Further analysis showed that melatonin (at 100 mg/kg) reduces X-ray irradiation-induced DNA damage by upregulation the expression of DNA repair genes (*Ogg1*, *Apex1*, and *Xrcc1*) [43]. Additionally, pretreatment of irradiated rats with melatonin can ameliorate the oxidative damage induced by ionizing radiation [44]. In another study melatonin promoted porcine somatic cell nuclear transfer by protecting embryonic cells from oxidative stress-induced DNA damage [45]. Such protective effect of melatonin was also observed against bisphenol A [46], formaldehyde [47], and phenytoin sodium-induced DNA damage [48].

Recent studies summarize the DNA protective mechanisms of melatonin as an indirect antioxidant agent. These mechanisms include: enhancing the activity of mitochondrial electron transport chain, pro-oxidative enzymes inhibition, augmentation of other antioxidant agents activity, glutathione synthesis stimulation, and antioxidant enzymes protection and activation [49]. Normalizing mitochondrial function was also investigated to target cancer cells. A decrease in ATP production and viability was observed in breast cancer MCF-7 cells after treatment with melatonin [50]. More analysis of the role of melatonin in mitochondrial function showed an up-regulation in complex III activity after treatment with the main melatonin catabolite 6-hydroxymelatonin (6-OHM). Additionally, melatonin and its main metabolite (6-OHM) can induce cell death in breast MCF-7 and leukemic HL-60 cells by increasing the production of reactive oxygen species (ROS) including H_2_O_2_ [51]. Furthermore, the combination of melatonin and rapamycin induced changes in mitochondrial functions that resulted in increased ROS, apoptosis and mitophagy [52]. Similarly, melatonin can reduce liver damage by recovering mitochondrial mitophagy [53]. The combined effect of these mechanisms makes melatonin an important agent to protect DNA from oxidative stress.

DNA repair system was also evaluated as a target for melatonin. In one study, melatonin enhanced DNA repair capacity in MCF-7 (breast cancer) and HCT-15 (colon cancer) cell lines by affecting genes involved in DNA damage responsive pathways [54]. Such activation in the DNA repair system was also reported for melatonin against DNA damage induced by hydrogen peroxide [40].

Telomerase is an enzyme involved in telomere elongation during cell division. The activity of this enzyme is essential for cancer cells as these cells have the capacity for sustained cell division and DNA replication. The effect of melatonin on telomerase activity was studied in vitro and on nude mice transplanted with MCF-7 xenograft. Results of this study showed a significant dose-dependent inhibition of telomerase activity in vivo and in vitro [55]. Additionally, melatonin delays ovarian aging by inhibiting telomerase activity [56].

### 2.2. Role of Melatonin in Sustained Proliferative Signaling

Cancer development and progression is directly associated with the ability of sustained proliferation. This is manifested by the presence of altered expression of proteins and signaling pathways related to cell cycle in cancer cells. Different signaling pathways were suggested as targets to inhibit sustained proliferation in cancer. These pathways include: signaling pathways of hypoxia-inducible factor-1 (HIF-1), NF-κB s, PI3K/Akt, insulin-like growth factor receptor (IGF-1R), cyclin-dependent kinases (CDKs), and estrogen receptor signaling [13]. The effect of melatonin on most of these signaling pathways was evaluated by different studies.

HIF-1 is a heterodimer that responds to changes in oxygen levels. Under low oxygen concentration, this molecule enters the nucleus and stimulates the transcription of many genes responsible for tumor aggressiveness [57]. A recent study showed a reduction in HIF-1 levels after melatonin treatment in serous papillary ovarian carcinoma of ethanol-preferring rats [58]. The antioxidant activity of melatonin against reactive oxygen species caused destabilization of HIF-1α protein levels and suppresses its transcriptional activity in the HCT116 human colon cancer cells [59].

Further analysis of melatonin’s destabilizing effect against HIF-1α revealed the involvement of phingosine kinase 1 (SPHK1) which is a new HIF-1α modulator. Melatonin reduces SPHK1 activity in PC-3 prostate cancer cells under hypoxic condition. Additionally, melatonin inhibits the Akt/glycogen synthase kinase-3β (GSK-3β) signaling pathway which stabilizes HIF-1α [60]. Similar HIF-1 inhibitory effects of melatonin were observed in other cancer models, including pancreatic ductal cells, cervical cancer, and lung cancer [61]. Melatonin was also effective through HIF-1 inhibition [62].

Nuclear factor-kappaB (NF-κB) is a family of transcription factors involved in many cellular pathways leading to inflammation and immune response [63]. An anti-inflammatory response of melatonin was reported by NF-κB inhibition [64]. Also melatonin inhibited the nuclear translocation of NF-κB to enhance the anticancer effect of berberine against lung cancer [65]. Additionally, melatonin mediated NF-κB inhibition prevented motility and invasiveness in HepG2 liver cancer cells [66] and protected against cyclophosphamide-induced urinary bladder injury [67]. 

Phosphoinositide 3-kinase (PI3Ks) is a family of lipid kinases that is involved in regulation of many cellular mechanisms including cell proliferation and differentiation [68]. Several studies reported the inhibitory effect of melatonin on PI3K signaling pathway. The proliferation of MDA-MB-361 breast cancer cells was suppressed after melatonin treatment through inhibition of PI3K/Akt signaling pathway [69]. A combination of melatonin with vitamin D3 caused inhibition in proliferation of MCF-7 breast cancer cells by downregulation of Akt expression [70]. Additionally, melatonin combined with endoplasmic reticulum stress-inducers (thapsigargin or tunicamycin) caused cell death in melanoma cells by inhibiting the PI3K/Akt/mTOR pathway [71].

Cyclin-dependent kinases (CDKs) are enzymes essential for cell division and transcription. They are serine/threonine kinases that are important for cancer progression [72]. The inhibitory effect of melatonin on CDKs was explored in several studies. In one study, melatonin (at millimolar concentrations) inhibited the proliferation of rat dopaminergic neuroblastoma cells by suppressing the progression of the G1-phase. This suppression was mediated by inhibiting the transcriptional activity of cyclins and CDKs [73]. Downregulation of cyclin D1, CDK4, cyclin B1 and CDK1 was reported as antiproliferative mechanism of melatonin against human osteosarcoma cell proliferation [74]. Cyclin D1 was also inhibited after treating breast cancer cells with melatonin [75].

The role of estrogen in mammary cancer development is well studied. The hormone may stimulate the proliferation of mammary cancer cells through stimulation of estrogen receptors. This stimulation can propagate the number of mutations induced by different carcinogens [76]. Catechol-estrogens are estrogenic metabolites that have direct mutagenic effect on DNA through generation of free radicals after oxidation of these metabolites [77]. Melatonin can alter the effect of estrogen in three different ways: (1) Inhibition of steroid synthesis by gonads; (2) downregulation of the synthesis of enzymes involved in androgen synthesis such as aromatase; (3) binding with estrogen receptor to inhibit its stimulatory effect [78].

### 2.3. Role of Melatonin in Evasion of Anti-Growth Signaling

Evasion of antigrowth signals is an essential step for cancer cells to continue to proliferate. Cancer cells need to inhibit tumor suppressor genes that are responsible for antigrowth signals. Mutations in tumor suppressor genes were observed in cancer cells with the most frequently mutated tumor suppressor gene is p53 followed by phosphatase and tensin homolog (PTEN), adenomatous polyposis coli (APC), ataxia-telangiectasia mutated (ATM), breast cancer gene2 (BRCA2), Von Hippel-Lindau (VHL), retinoblastoma (RB), cyclin-dependent kinase inhibitor 2A (CDKN2A), breast cancer gene2 (BRCA1) and Wilms tumor (WT1). Mutant p53 was reported in more than 50% of all tumors [13]. The expression of p53, BRCA1, BRCA2 was increased in breast cancer cells after treatment with melatonin [79]. Further analysis of the effect of melatonin revealed the ability of this hormone to induce phosphorylation of p53 at Ser-15 causing proliferation inhibition and prevention of DNA damage accumulation [80]. Increased p53 expression was also observed in prostate cancer cells following treatment with melatonin [81].

### 2.4. Role of Melatonin in Resistance to Apoptosis

Cancer cells can evade apoptosis by overexpressing anti-apoptotic proteins that inhibit the process of apoptosis. Several pathways are responsible for apoptosis evasion in cancer. Overexpression of molecules that resist apoptosis is a main strategy used by cancer cells to avoid apoptosis [13]. B-cell lymphoma-2 (Bcl-2) family has an important role in apoptosis resistance. Treatment of pancreatic cancer cells with melatonin caused an induction of apoptosis mediated by down-regulation of Bcl-2 and up-regulation of Bax (pro-apoptotic protein) [82]. Modulation of Bcl-2/Bax was also reported as a mechanism of apoptosis induction by melatonin against human pancreatic carcinoma cells [83]. Similar results were obtained for human myeloid leukemia cells treated with melatonin which inhibit the progression from G1 to S phase of the cell cycle by Bax up-regulation and Bcl-2 down-regulation [84]. Additionally, treatment of ovarian cancer cells with melatonin decreased cell proliferation by increasing the number of cells in G1 phase and decreasing the number of cells in S phase [52].). Other studies have explored various targets of melatonin to induce apoptosis. These targets include upregulation of pro-apoptotic (p53, Bax, total and cleaved caspase-3) and anti-apoptotic (Bcl-2 and survivin) proteins in addition to downregulation of cyclin dependent kinases [34,85]. The apoptosis induction effect of melatonin was also reported in other cancer models including human hepatoma [86], murine gastric cancer [87], and prostate cancer [81].

### 2.5. Role of Melatonin in Replicative Immortality

Replicative immortality is the ability of cells to divide continuously. This capacity is a characteristic feature for cancer cells that can undergo unlimited cycles of cell division. This process can be repressed by inhibiting different targets including telomerase, mammalian target of rapamycin (mTOR), CDK4/6, CDK 1/2/5/9, Akt and PI3K [13].

The effect of melatonin on CDKs and Akt/PI3K pathways were discussed in a previous section (sustained proliferation signaling). Telomerase is a specialized DNA polymerase that extends the ends of shortening chromosomes in dividing cells. Without the activity of this enzyme, the chromosomes will be unstable and most types of cancer depend on activation of telomerase to maintain continuous cell division [88]. Exposure of breast cancer cells to increasing concentrations of melatonin caused a dose-dependent decrease in telomerase activity in vitro and in vivo [89]. In another study, melatonin receptor agonists inhibited the expression of human catalytic subunit of telomerase [90]. Furthermore, combination of melatonin with *cis*-diamminedichloroplatinum inhibited human ovarian cancer by lowering telomerase activity. This inhibition was significantly higher than that observed in single treatment groups [91]. Additionallytelomerase inhibition was also reported in more recent studies [92].

The importance of the PI3K/AKT/mTOR signaling pathway was reported in different cancer types and its activation is associated with advanced tumor stage and poor prognosis [93]. Mammalian target of rapamycin (mTOR) is a serine-threonine protein kinase that is involved in regulation of many physiological pathways, including cell growth, proliferation, metabolism, protein synthesis, and autophagy [94]. Treatment of tumor bearing rats with melatonin for 60 days resulted in reduction in tumor size associated with decreased levels of mTOR compared with the negative control [95]. In another study, melatonin in combination with arsenic trioxide inhibited human breast cancer cells by reducing mTOR expression in treated cells [96]. In another study, melatonin combined with rapamycin suppressed AKT/mTOR pathway in head and neck cancers [52]. Additionally, melatonin decreased H_2_O_2_-induced phosphorylation of mTOR in hepatoma cells [97].

### 2.6. Role of Melatonin in Tumor Dysregulated Metabolism

Increased glucose uptake and lactate production (the Warburg effect) is a characteristic feature of many cancer cells [98]. To achieve this altered metabolism, active oncogenes and inactive tumor suppressor genes in cancer cells resulted in a change in the expression and activity of several components in glucose and glutamate metabolism. Several glycolytic enzymes are key regulators for cancer dysregulated metabolism. Enzymes like hexokinase2 (HK2), 6-phosphofructo-2-kinase/fructose-2,6-biphosphatase 3(PFKFB3) and pyruvate kinase isoform M2 (PKM2) are suitable targets to inhibit cancer metabolism [13].

Several transcriptional factors are involved in the establishment of the Warburg effect in cancer cells. Hypoxia-inducible factor 1 (HIF1) is one of these factors and it enhances the expression of most glycolytic enzymes and glucose transporters. HIF1 also upregulates expression of pyruvate dehydrogenase kinases (PDKs) which inactivates pyruvate dehydrogenase by phosphorylation. Inactive pyruvate dehydrogenase will stop the conversion of pyruvate to acetyl-CoA and increase lactate production [98]. Melatonin has an inhibitory effect on HIF1 which in turn inhibit the altered metabolism of cancer. In the previous section (sustained proliferative signaling) the inhibition of HIF1 by melatonin was discussed [57,58,59,60,61,62].

Another metabolic regulator of cancer metabolism is *MYC* oncogene. Overexpression of *MYC* in cancer cells cause upregulation of many genes including glycolytic enzymes, PDK1, lactate dehydrogenase, and glucose transporters [99]. Lactate dehydrogenase inhibition and a reduction in lactate were observed in different cell lines after treatment with melatonin [100]. Downregulation of MYC oncogene and upregulation of pro-apoptotic genes (*BAD* and *BAX*) were observed in breast cancer cells treated with melatonin [101]. Additionally, combination of melatonin and sorafenib cause down-regulation of MYC oncogene in hepatocellular carcinoma [102].

### 2.7. Role of Melatonin in Tumor-Promoting Inflammation

The link between chronic inflammation and cancer development was reported in previous studies [103,104]. Inflammation can directly contribute to carcinogenesis by generating different carcinogenic products including reactive oxygen species and reactive nitrogen species which can induce DNA damage and cancer development [105]. Many factors are important targets that can be modulated to control inflammation damaging effects. These factors include cyclooxygenase-2 (COX-2), NF-κB, tumor necrosis factor alpha (TNF-α), and inducible nitric oxide synthase (iNOS) [13]. Melatonin (10 mg/Kg) was used to treat chronic bowel inflammation in rat models of colitis. Results showed a decrease in the inflammation mediated by local inhibition of iNOS and COX-2 expression in colonic mucosa [106]. Further testing revealed that melatonin inhibition of iNOS and COX-2 is through suppressing of p52 acetylation, binding, and transactivation [107]. In another study, melatonin inhibited both COX-2 expression and NF-κB activation in murine macrophage-like cells [108]. The up-regulation in the expression of the proapoptotic protein Bim and down-regulation of COX-2 expression were reported as a mechanism of action of melatonin to inhibit in human breast carcinoma MDA-MB-231 cells [109].

### 2.8. Role of Melatonin in Angiogenesis Inhibition

Angiogenesis (blood vessel formation) is an essential process in cancer development and progression as it provides dividing cells with the oxygen and nutrients needed to sustain cell division [110]. Tumor cells stimulate angiogenesis by activating angiogenic factors and inhibiting factors that stop angiogenesis [111]. The main angiogenic factors include vascular endothelial growth factor (VEGF), platelet-derived growth factor (PDGF), epidermal growth factor (EGF), and hepatocyte growth factor (HGF) [112]. The expression of VEGFR-2 and micro-vessels density was inhibited in mice treated with melatonin [113]. Disruption of tumor blood vessels formation was also observed in renal adenocarcinoma mouse model treated with melatonin [114]. The inhibitory effect of melatonin on serum VEGF levels was reported in previous studies [115,116]. A clinical study showed that oral administration of melatonin reduced serum VEGF levels in patients having cancer metastasis [117]. Additionally, the level of secreted VEGF and its mRNA were decreased in pancreatic carcinoma cells treated with melatonin for 24 h [118]. The inhibitory effect of melatonin on VEGF was also observed in MCF-7 breast cancer cell line and glioblastoma cells [119,120]. Melatonin was also successful in augmenting the antiangiogenic activity of other agents used to inhibit VEGF expression. In one study a combination consisting of melatonin and *Propionibacterium acnes* showed reduction in VEGF serum levels and regression in tumor size in mice bearing breast carcinoma [27]. Additionally, combination of melatonin with pitvastatin caused 42% reduction in the levels of VEGF in the combination group compared with pitvastatin single therapy in rats with breast cancer [121].

Endothelial cell migration, invasion, and tube formation are essential steps in angiogenesis [116]. The effect of melatonin on endothelial cell migration, invasion and tube formation was evaluated in many studies. Melatonin caused a 32% inhibition in cell migration of human umbilical vein endothelial cells (HUVEC) and 50% inhibition in cell invasion and tube formation [122]. The reduction in migration and invasion of HepG2 cells was achieved after treatment with 1 mM melatonin for 48 h [115]. Further analysis of the mechanism of action of melatonin in inhibition of cell migration and invasion revealed that the action of this hormone involves inactivation of MMP-2 and MMP-9 in addition to down-regulation of p38 signaling pathway [123]. Additionally, melatonin inhibits hypoxia induced cell migration by inhibiting ERK/Rac1 pathway [124]. Another mechanism of angiogenesis suppression of melatonin is mediated by the inhibition of endothelin-1 which is a peptide produced by solid tumors to promote proliferation, metastasis, and angiogenesis [125].

### 2.9. Role of Melatonin in Tissue Invasion and Metastasis

The highest percentage of cancer mortality is reported in patients with metastatic cancers [126]. Cancer metastasis requires many steps, including loss of cell-cell contact, tissue invasion, intravasation, transport around the body, extravasation at the secondary site and establishment of a secondary tumor [127].

The disruption of cell–cell adhesion enables cancer cells to leave the primary tumor mass and invade surrounding tissue. Tight junctions, adherens junctions, gap junctions, desmosomes, and hemidesmosomes are the main cell-cell adhesion molecules [128]. Previous studies showed that melatonin has an inhibitory effect on invasive properties of cancer by altering the expression of tight and adherens junction proteins. E-cadherin is an important protein in tight junctions and its low expression was observed in metastatic cancers [129]. Increased E-cadherin expression was observed in breast cancer cells treated with melatonin [130]. A recent study provided additional details about the mechanism of action of melatonin in upregulating E-cadherin expression. Interference in the interaction between C/EBPβ and NF-κB is induced by melatonin and caused upregulation in E-cadherin expression [131]. Occludin is a trans-membrane protein present in tight junctions and is essential for normal function of this adhesion molecule [132]. The migration of human lung adenocarcinoma cell line A549 was inhibited after treatment with melatonin. This inhibition is mediated by upregulation of occludin expression [133]. Another example of molecules involved in metastasis is integrin. Integrin is a glycoprotein involves in linking the intracellular actin cytoskeleton with the extracellular matrix. Treatment of glioma cells with melatonin inhibited their invasion by modulating integrin expression [134]. Additionally, the in vitro invasive capacity of breast cancer cells was inhibited by melatonin through alteration of integrin expression [130].

Epithelial–mesenchymal transition (EMT) is an important step in cancer metastasis. During this process, cancer cells lose their adhesion to neighboring cells and become migratory [128]. Interference with NF-κB signaling pathway was reported as a mechanism of action of melatonin to inhibit EMT [113]. Vimentin is a cytoskeletal protein that is important for cell migration and maintenance of mesenchymal phenotype [135]. Treatment of breast cancer cells with melatonin caused a decrease in the expression of vimentin and inhibition of cell migration [136].

### 2.10. Role of Melatonin in Tumor Associated Immune Evasion

Cancer cells can escape from the immune system using different mechanisms including activation of regulatory cells, defective antigen presentation, immune suppression, and immune deviation [13]. The role of melatonin in activation of anticancer immune response was explored in different studies. Murine foregastric carcinoma cells were treated in vitro and in vivo with melatonin. In this study, melatonin caused dose dependent inhibition of tumor weight and volume and decreased the number of regulatory T cells infiltration. Also the expression of regulatory protein Foxp3 in regulatory T cells was inhibited by melatonin [137]. A shift in the immune response toward Th1 anticancer response was observed in tumor bearing mice treated with melatonin [27]. Similar results were observed when melatonin combined with thymoquinone to treat breast cancer in mice [25]. Additionally, melatonin potentiates the antitumor effect of IL-2 against melanoma [138]. In this study, an increase in the number of monocytes and natural killer cells was observed within 7–14 days of melatonin treatment [139]. Also the production of inflammatory cytokines was increased in monocytes and macrophages treated with melatonin [140]. Reactive oxygen species production and enhanced phagocytic activity were also reported in macrophages treated with melatonin [141].

Natural killer (NK) cells have an important role in the control of virally infected and cancer cells. Administration of melatonin increases NK cells number and activity [142]. This increase in the number of NK cells is a result of melatonin activation of T helper cells to produce several cytokines including IL-2, IL-6, IL-12 and interferon gamma (IFN-γ) [143]. T lymphocytes have melatonin receptors which explain the effect of melatonin on these cells to produce different cytokines that activate various cells including NK [144].

### 2.11. Melatonin Contradictory Effects

A large number of studies have proved the anticancer effects of melatonin. However, dual effects of this hormone were also reported. Such effects can be observed in the generation and inhibition of oxidative stress. The contribution of ROS in cancer development was studied extensively. Increased mutation rate, growth receptors activation, enhanced oncogenesis signaling, and angiogenesis promotion were observed in cells exposed to high ROS [145]. On the other hand, oxidative stress can inhibit cancer cell survival by induction of DNA damage, telomeres shortening, and oxidation of biological molecules [146]. 

Melatonin is very effective antioxidant by directly quench free radicals, production of active scavenger metabolites, increasing the expression of anti-oxidant enzymes, chelating metals and stabilizing mitochondria [147]. However, the antitumor effect of this indolamine is not always associated to its antioxidant activity. Recent studies showed that melatonin antitumor effects can be achieved by stimulating ROS production [148,149] which is exactly the opposite of its effect as antioxidant. Another example of the dual effect of melatonin is reflected by its behavior as pre-apoptotic and anti-apoptotic agent. Anti-apoptotic effect of melatonin was observed in normal cells exposed to toxic or metabolic injury [150,151]. This effect is not limited to normal cells and an increase in the expression of genes associated with cell survival was also reported in glioma cells treated with melatonin [152]. On the other hand, many studies proved the apoptosis induction effect of melatonin against many cancer types including gastric and cervical cancers [153,154].

The paradoxical action of melatonin in cancer treatment requires further research to be fully understood. The majority of researches showed that physiological concentrations (nanomolar) of melatonin can only induce cytostatic effect; while apoptosis induction effect can be achieved at higher concentrations (millimolar) [155].

One explanation of the contradictory results of melatonin is the difference in experimental procedures and cancer models used in the different studies. Differences in incubation conditions, treatment duration, and passage number of treated cells (continuous sub-culturing may alter the expression of melatonin receptors) can cause a difference in response toward melatonin treatment [156]. The selective killing of cancer cells by melatonin make this hormone acting like a smart killer by recognizing its location context (normal or cancer) and selecting the suitable action. Further research is needed to identify the factors that help melatonin to recognize the context and to produce the correct response.

## 3. Conclusions

**Figure 2:**
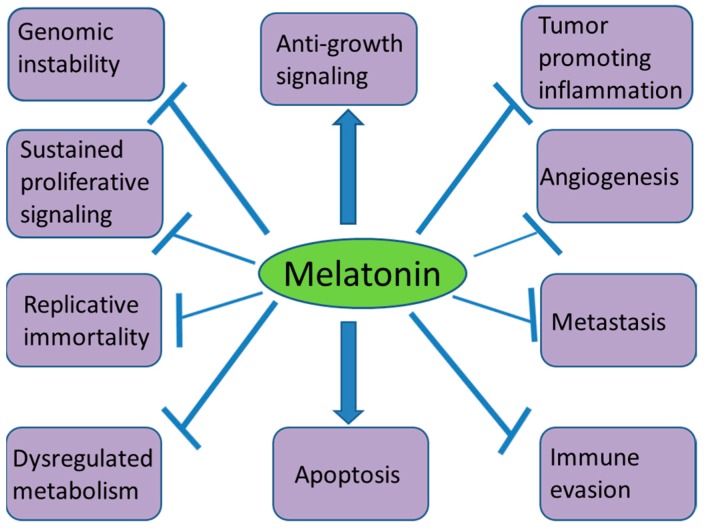
Effects of melatonin on different cancer hallmarks. 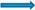 stands for stimulation; 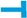 stands for inhibition.

The anticancer activity of melatonin has been reported in many experimental and clinical studies. The inhibitory effects of this hormone can be achieved as a single therapeutic agent or in combination with other therapies. The involvement of melatonin in activating various anticancer mechanisms in different cancer hallmarks (Figure 2) makes this molecule an important physiological anticancer agent. More clinical studies are needed to consider melatonin as a standard therapeutic option to treat some cancers.
